# Acculturative stress and problematic smartphone use among international students in Japan: a sequential mediation model of emotion regulation and social anxiety

**DOI:** 10.3389/fpubh.2026.1860115

**Published:** 2026-08-14

**Authors:** Guobao Wang, Lei Gao, Nobuo Watanabe

**Affiliations:** 1 Department of Economics and Management, School of Economics and Management, Baoshan University, Baoshan, Yunnan, China; 2 Department of Management Information Studies, Graduate School of Management and Information Studies, Josai International University (Josai Kokusai Daigaku), Togane, Chiba, Japan

**Keywords:** acculturation, anxiety, emotional regulation, international students, Japan, smartphone, technology addiction

## Abstract

**Background:**

Problematic smartphone use (PSU) is an increasing public health concern among international students and may be linked to acculturative stress during cross-cultural adaptation. However, the psychological mechanisms underlying this association remain insufficiently understood. This study examined the association between acculturative stress and PSU among international students in Japan and tested the independent and sequential mediating roles of emotion regulation strategies and social anxiety.

**Methods:**

A cross-sectional survey was conducted between March and September 2025 among 732 international students in Japan. Participants completed self-report measures of acculturative stress, cognitive reappraisal, expressive suppression, social anxiety, and PSU. A conceptual model comprising six hypotheses was tested using Pearson correlation analysis, confirmatory factor analysis, and structural equation modeling. Indirect effects were evaluated using 5,000 bootstrap resamples.

**Results:**

The prevalence of PSU was 48.9%. Acculturative stress was positively associated with PSU (direct effect = 0.089, *p* < 0.001). Cognitive reappraisal (indirect effect = 0.043, *p* < 0.001), expressive suppression (indirect effect = 0.017, *p* < 0.001), and social anxiety (indirect effect = 0.033, *p* < 0.001) each significantly mediated this relationship. In addition, significant sequential indirect effects were observed through cognitive reappraisal followed by social anxiety, and through expressive suppression followed by social anxiety. The total indirect effect accounted for 58.3% of the total association between acculturative stress and PSU. Overall, the findings supported all six hypotheses proposed in the conceptual model.

**Conclusion:**

The empirical testing and support of the proposed sequential mediation model represent an important contribution of this study and provide a novel framework for understanding the psychosocial correlates of PSU. The findings underscore the public health relevance of acculturative stress among international students and suggest that adaptive emotion regulation and social anxiety may be important targets for prevention. Universities and public health practitioners should consider culturally sensitive strategies to support international students' mental well-being and digital health behaviors.

## 1 Introduction

International student mobility has increased substantially, and Japan has become an important destination for students pursuing education abroad. According to the most recent survey by the Japan Student Services Organization, Japan hosted 408,069 international students as of May 1, 2025 (1). Although studying abroad can provide important educational and intercultural opportunities, international students frequently encounter language barriers, unfamiliar social norms, academic demands, discrimination, homesickness, and limited social support. These experiences may generate acculturative stress and adversely affect psychological adjustment (2). A recent systematic review and meta-analysis confirmed that acculturative stress is consistently associated with poorer psychological outcomes among international students (3).

Smartphones play an important but potentially conflicting role in this population. They facilitate communication with family and friends, provide language and navigation assistance, and support access to academic and practical information in the host country (4, 5). However, reliance on smartphones for distraction, emotional relief, or avoidance may develop into problematic smartphone use (PSU), characterized by impaired control and interference with everyday functioning. Compensatory Internet Use Theory proposes that excessive digital engagement may function as a response to unmet psychosocial needs or offline distress rather than simply reflecting inadequate self-control (6). Consistent with this perspective, a study of international college students in the United States found that acculturative stress was associated with internet addiction (7). More recently, research among international students in China identified both direct and indirect associations between acculturative stress and smartphone addiction (8). Nevertheless, evidence remains limited among international students in Japan, and the psychological pathways underlying this association have not been adequately examined.

Emotion regulation (e.g., cognitive reappraisal and expressive suppression) may represent one such pathway (9). Cognitive reappraisal involves changing the interpretation of an emotional situation, whereas expressive suppression involves inhibiting the outward expression of emotion (10). Persistent acculturative demands may be associated with reduced use of cognitive reappraisal and greater reliance on suppression (11). These patterns may encourage individuals to use smartphones for distraction or short-term emotional relief (12). A recent systematic review and meta-analysis found that emotion dysregulation was positively associated with PSU and that expressive suppression also showed a positive, although weaker, association with PSU (13). Cognitive reappraisal and expressive suppression may therefore account for distinct portions of the association between acculturative stress and PSU.

Social anxiety may provide a further explanatory pathway. International students may fear negative evaluation or interpersonal mistakes when communicating in an unfamiliar linguistic and cultural environment (14). Smartphone-mediated communication can offer greater control and fewer immediate interpersonal demands than face-to-face interaction. Although this may provide temporary relief, repeated reliance on digital interaction may reinforce avoidance and increase vulnerability to PSU (15). A meta-analysis identified a significant association between social anxiety and mobile phone addiction (16), while more recent longitudinal evidence has further supported the close relationship between social anxiety and problematic mobile phone use (17). Acculturative stress may therefore be associated with PSU partly through elevated social anxiety.

Emotion regulation and social anxiety may also operate sequentially rather than independently. Individuals exposed to chronic acculturative stress may first experience difficulty regulating emotional responses, which may then increase vulnerability to social anxiety (18). For example, reliance on expressive suppression may heighten internal distress and reduce confidence in interpersonal situations, whereas lower use of cognitive reappraisal may limit adaptive coping and increase fear of negative evaluation (19). In turn, elevated social anxiety may encourage greater reliance on smartphone-mediated interaction as a way of avoiding uncomfortable face-to-face experiences. Although emerging evidence suggests that emotion regulation and anxiety may operate in sequence to shape maladaptive behavioral outcomes (20), this pathway has rarely been examined in relation to acculturative stress and PSU, particularly among international students in Japan.

**Figure 1:**
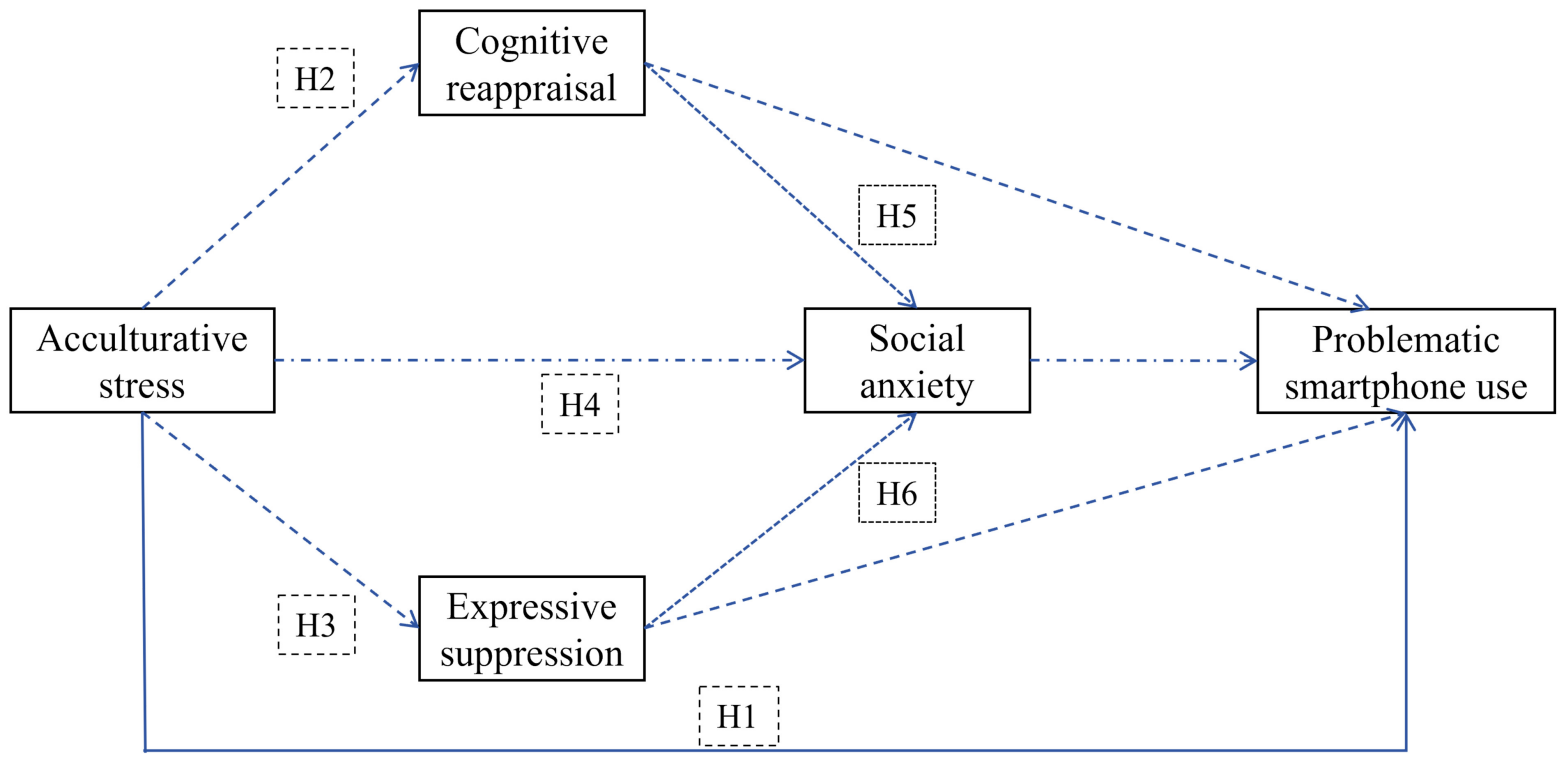
Conceptual diagram of the proposed chain mediation model.

Addressing this research gap, the present study investigates whether acculturative stress is associated with PSU among international students in Japan, and whether this association is sequentially mediated by emotion regulation strategies and social anxiety. Drawing on acculturation theory (2), emotion regulation theory (9), and Compensatory Internet Use Theory (6), we propose a conceptual model (Figure 1) testing six hypotheses: (H1) acculturative stress is positively associated with PSU; (H2–H4) cognitive reappraisal, expressive suppression, and social anxiety independently mediate this association; and (H5–H6) cognitive reappraisal and social anxiety, as well as expressive suppression and social anxiety, sequentially mediate the relationship between acculturative stress and PSU.

## 2 Materials and methods

### 2.1 Study design and participants

A cross-sectional investigation was conducted at universities in Tokyo and Chiba, Japan, between March and September 2025. Participants were recruited *via* convenience sampling, with the majority originating from Asian countries (e.g., China, Vietnam, Nepal, and South Korea). This demographic composition aligns closely with national statistics regarding international students in Japan. According to JASSO (2025) (1), approximately 93.4% of international students originate from the Asian region. Consequently, the current sample demonstrates high ecological validity regarding the international student population in the Japanese context. The eligibility criteria included: (1) age ≥ 18 years; (2) current enrollment as an international student; (3) smartphone ownership; (4) adequate cognitive and linguistic capacity to comprehend and complete the questionnaire; (5) self-reported absence of clinically diagnosed addictive disorders, affective disorders, or substance use disorders; and (6) provision of written informed consent for voluntary participation. Exclusion criteria were: (1) severe physical or psychiatric comorbidities; (2) withdrawal from participation during data collection; and (3) insufficient proficiency in English or Japanese to engage with study questionnaires. The reporting of this study follows the Strengthening the Reporting of Observational Studies in Epidemiology (STROBE) guidelines for cross-sectional studies.

### 2.2 Ethical approval

The study was approved by the Ethics Committee of Josai International University (Approval No. 17000096) and was conducted in accordance with the Declaration of Helsinki.

### 2.3 Sample size

The minimum required sample size was calculated using the formula for population proportion estimation in cross-sectional studies: *n* = Z^2*^ p(1–p) /E^2^ (21), where *Z* = 1.96 (corresponding to a 95% confidence level); *p* = 0.5 (a conservative prevalence estimate for PSU to maximize sample size); and *E* = 0.05 (acceptable margin of error). Substituting these values yielded a minimum sample size of 385. To account for potential data exclusion due to incomplete or inconsistent responses, the sample size was adjusted upward by 20%, resulting in a target of 482 participants. Furthermore, given the mediation analysis central to our study, we also conducted a power analysis for mediation effects based on the guidelines by Fritz and Mackinnon (22). According to their guidelines, detecting even small-sized mediated effects (α path and β path both at a small level, standardized coefficients = 0.14) requires a sample size of 462 using a bias-corrected bootstrap to achieve 80% power. Our final sample of 732 participants exceeds this requirement.

### 2.4 Measures

#### 2.4.1 Sociodemographic data

Sociodemographic characteristics, including age, gender, educational attainment, nationality, duration of study abroad, academic major, living arrangements, average daily smartphone usage, marital status, and Japanese language proficiency, presence of relatives in Japan, and living expense sources, were collected using a structured questionnaire administered during the survey. Age was collected in completed years and analyzed as a continuous variable. Daily smartphone use was assessed using three prespecified ordinal categories (< 2 h, 2–4 h, and ≥5 h per day), while duration of study abroad was assessed using three categories (< 1 year, 1–3 years, and >3 years). These categorical response options were specified in the questionnaire before data collection.

#### 2.4.2 Acculturative stress

Acculturative stress was assessed using the Acculturative Stress Scale for International Students (ASSIS) (23). The ASSIS is a widely established instrument, and previous studies involving international student populations have consistently confirmed its robust construct validity and high internal consistency (24, 25). Because a standardized Japanese version was not available, the authors translated the original scale using a rigorous forward-backward translation method, the details of the translation and adaptation procedures are provided in the “2.5 Data Collection Procedure” section. The ASSIS comprises 36 items across seven dimensions: perceived discrimination (8 items), homesickness (4 items), perceived hate/rejection (5 items), fear (4 items), stress due to culture shock/change (3 items), guilt (2 items), and nonspecific concerns (10 items). To enhance contextual relevance, minor wording modifications were made. For instance, the original item “I feel nervous to communicate in English” was revised to “I feel nervous to communicate in Japanese” to reflect the primary language environment of the host institution. Responses were rated on a 5-point Likert scale ranging from 1 (“strongly disagree”) to 5 (“strongly agree”), yielding a total score ranging from 36 to 180, with higher scores indicating greater difficulties in cultural adaptation. In this study, the Cronbach's α coefficients for the seven subscales of the ASSIS ranged from 0.721 to 0.900.

#### 2.4.3 Emotion regulation strategies

The Emotion Regulation Questionnaire (ERQ) was employed to assess participants' use of two emotion regulation strategies: cognitive reappraisal and expressive suppression (9). To ensure linguistic suitability for the study context, we utilized the validated Japanese version (ERQ-J) developed by Yoshizu et al. (26). Previous research has confirmed that the ERQ-J maintains the original two-factor structure and demonstrates adequate internal consistency as well as test-retest reliability (26, 27). The ERQ comprises 10 items divided into two subscales: cognitive reappraisal (ERQC, 6 items) and expressive suppression (ERQE, 4 items). Responses are recorded on a 7-point Likert scale ranging from 1 (“strongly disagree”) to 7 (“strongly agree”). Total scores for cognitive reappraisal and expressive suppression range from 6 to 42 and 4 to 28, respectively, with higher scores indicating greater habitual use of each strategy. In this study, the Cronbach's α coefficients were 0.864 for cognitive reappraisal and 0.846 for expressive suppression.

#### 2.4.4 Social anxiety

Social anxiety symptoms were evaluated using the Liebowitz Social Anxiety Scale (LSAS) (28). To ensure cultural and linguistic appropriateness for the study population in Japan, the validated Japanese version (LSAS-J) was also utilized (29). The LSAS-J has demonstrated good psychometric properties in previous research, with studies reporting high internal consistency, strong test-retest reliability, and confirmed factorial and convergent validity (29, 30). This 24-item instrument assesses social anxiety across two subscales: fear (11 items) and avoidance behavior (13 items). Participants rated fear-related items on a 4-point Likert scale from 0 (“none”) to 3 (“severe”) and avoidance-related items from 0 (“never”) to 3 (“usually”). Total scores range from 0 to 72, with higher scores indicating more severe social anxiety. In this study, the Cronbach's α coefficients for the two subscales of LSAS were 0.927 and 0.937, respectively.

#### 2.4.5 PSU

The short version of the Smartphone Addiction Scale (SAS-SV) was used to measure the levels of smartphone addiction (31). The reliability and validity of the SAS-SV have been well-documented, with studies confirming that the scale demonstrates high internal consistency and robust concurrent validity (31, 32). The scale comprises 10 items rated on a 6-point Likert scale (1 = strongly disagree, 6 = strongly agree). Total scores range from 10 to 60, with higher scores indicating a greater tendency toward smartphone addiction. Since a standardized Japanese version was not available for this study, the original English scale was translated. During the translation process and following the precedent set by previous Japanese studies (33, 34), the application “LINE” was explicitly added to item 8 (“Constantly checking my smartphone so as not to miss conversations between other people on Twitter or Facebook”) to reflect the dominant social network habits in Japan. Gender-specific cut-off scores were applied to identify high-risk smartphone addiction: 31 for males and 33 for females (31). In this study, the Cronbach's α coefficient for the SAS-SV was 0.951.

### 2.5 Data collection procedure

To facilitate participation among students with proficiency in either English or Japanese, a bilingual questionnaire was used. The survey adopted a parallel-display format, in which each item was presented first in English and then in Japanese. English was selected as the primary language because it is commonly used as a lingua franca among international students from diverse linguistic backgrounds. Where validated Japanese versions of the study measures were available, these were used preferentially. Specifically, the study adopted the published Japanese versions of the ERQ (26) and the LSAS (29). The use of established Japanese versions supported linguistic appropriateness and consistency with prior research.

For the Acculturative Stress Scale for International Students (ASSIS) and the Smartphone Addiction Scale-Short Version (SAS-SV), no standardized and publicly available Japanese versions were identified. Therefore, these two measures were translated using a forward-backward translation procedure (35). First, a bilingual researcher with expertise in psychology translated the original English scales into Japanese. Next, an independent bilingual expert, who was blinded to the original wording, back-translated the Japanese versions into English. Discrepancies between the original and back-translated versions were reviewed and discussed by a panel of three researchers until consensus was reached. The translated ASSIS and SAS-SV were then pilot-tested with 50 international students to assess clarity, comprehensibility, and cultural appropriateness. Minor wording revisions were made on the basis of participant feedback to improve readability. The final English-Japanese bilingual questionnaire was subsequently used for data collection.

Data collection was conducted between March and September 2025 using a paper-based survey administered to international students in Tokyo and Chiba. The research team collaborated with the International Student Research Association and student union leaders at several universities to facilitate participant recruitment. Questionnaires were distributed during breaks between classes based on institutional schedules. To reduce social desirability bias and protect privacy in this face-to-face setting, participants returned completed questionnaires anonymously using sealed envelopes or collection boxes.

A total of 816 questionnaires were distributed, and 773 were returned. After data screening, 41 responses were excluded because of invalid response patterns, including invariant responding (straight-lining) and other indicators of low-quality responding. The final sample comprised 732 valid responses, yielding a valid response rate of 89.7%.

### 2.6 Statistical analysis

All statistical analyses were conducted using SPSS 27.0 (IBM Corporation, Armonk, NY, USA). Descriptive statistics including frequencies and percentages were calculated for categorical variables, while means and standard deviations (SD) were used to summarize continuous variables. Differences in continuous variables between the PSU and non-PSU groups were examined using independent-samples *t*-tests and differences in categorical variables were examined using Pearson's chi-square tests. To compare continuous SAS-SV scores across the participant characteristics presented in the violin plots, independent-samples *t*-tests were used for variables with two categories, including age group and gender. One-way analysis of variance (ANOVA), followed by Tukey's honestly significant difference *post hoc* test, was used for educational attainment and daily smartphone use duration. Homogeneity of variance was assessed using Levene's test, and the assumption was satisfied for both three-category variables. For visualization in the violin plots, age was categorized as < 22 and ≥22 years because 22 years approximately corresponds to the completion of a conventional four-year undergraduate program in Japan. Age was retained as a continuous variable in the other statistical analyses. Bivariate relationships between variables were assessed using Pearson correlation analysis. Multicollinearity among explanatory variables was deemed negligible, as all variance inflation factor (VIF) values remained below the recommended threshold of 5 (36). For the estimation of PSU prevalence and comparisons between the PSU and non-PSU groups, PSU was treated as a binary variable based on the gender-specific SAS-SV cut-off scores of ≥31 for males and ≥33 for females. In contrast, the total SAS-SV score was treated as a continuous variable in the Pearson correlation analysis and mediation analysis.

Because all primary variables were assessed using self-report questionnaires, Harman's single-factor test was performed to examine whether a dominant single factor accounted for a substantial proportion of the observed variance. The first factor accounted for 34.67% of the total variance, well below the critical threshold of 40% (37), indicating that a dominant single-factor pattern was not evident.

Structural equation modeling (SEM) was performed *via* AMOS 26.0 (IBM Corporation, Armonk, NY, USA). Following the two-step approach recommended by Anderson and Gerbing (38), we first tested the measurement model using confirmatory factor analysis (CFA) to establish the construct reliability and validity, and subsequently examined the structural model to test the hypothesized relationships. For the measurement model, acceptable model fit was indicated by the following indices: a chi-square/degrees of freedom (χ^2^/df) ratio < 3, root mean square error of approximation (RMSEA) ≤ 0.08, standardized root mean square residual (SRMR) ≤ 0.08, goodness of fit index (GFI) ≥ 0.90, comparative fit index (CFI) ≥ 0.90, Tucker-Lewis index (TLI) ≥ 0.90, and normed fit index (NFI) ≥ 0.90 (39). Convergent validity was evaluated through factor loadings (≥ 0.50), composite reliability (CR ≥ 0.70), and average variance extracted (AVE ≥ 0.50). Discriminant validity was assessed using the Heterotrait-Monotrait (HTMT) ratio of correlations, with values below 0.85 considered indicative of distinct constructs (40). The model specified acculturative stress as the independent variable, PSU as the dependent variable, and emotion regulation strategies and social anxiety as sequential mediators.

Furthermore, to ensure the robustness of the structural model, demographic variables that were significantly associated with PSU in the baseline comparative analysis (i.e., gender, age, educational attainment, and daily smartphone use) were included as covariates in the final SEM. Standardized path coefficients (β), together with their corresponding standard errors (SEs), *p* values, and 95% confidence intervals (CIs), were reported to facilitate comparisons of the relative strengths of the structural relationships. Total, direct, and indirect effects were reported as unstandardized estimates (B). The significance of indirect effects was assessed using 5,000 bootstrap resamples to generate bias-corrected 95% CIs, with an effect considered statistically significant when the corresponding CI did not include zero. Statistical significance for all analyses was set at a two-tailed *P* value < 0.05.

## 3 Results

### 3.1 Characteristics of participants

**Figure 2:**
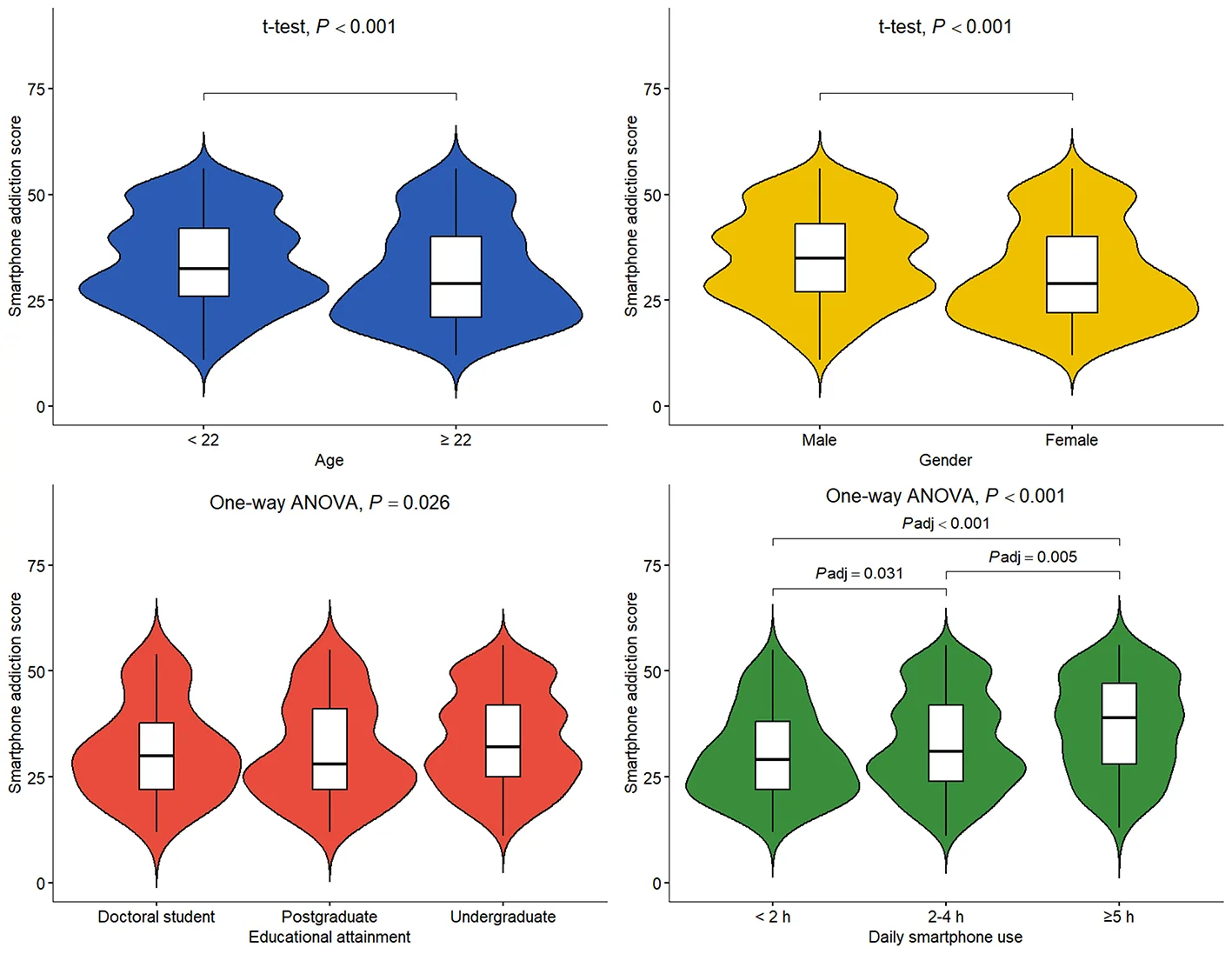
Differences in problematic smartphone use (PSU) scores according to participant characteristics. Violin plots show score distributions, with embedded box plots representing the median and interquartile range. Overall significance levels for *t*-tests and one-way ANOVA are shown at the top of each panel. Horizontal brackets indicate significant *post hoc* pairwise comparisons, where *P*adj denotes Tukey-adjusted *p*-values. Notably, no pairwise difference in educational attainment was significant despite a significant overall ANOVA result.

The detailed socio-demographic characteristics of the participants are presented in Table 1. The final sample consisted of 732 international students, with a mean age of 22.98 years (range: 18–31 years), and a nearly equal gender distribution (47.5% male, 52.5% female). The majority were enrolled in undergraduate programs (72.3%), lived independently (88.0%), and originated from East and Southeast Asia (94%), including China, South Korea, and Thailand. Based on the cut-off criteria of the SAS-SV, 48.9% of participants met the threshold for PSU. As shown in Figure 2, PSU scores differed significantly across age groups, gender, educational attainment levels, and daily smartphone use categories. In particular, PSU scores differed significantly across educational attainment groups at the omnibus level, although Tukey's HSD *post hoc* comparisons did not reveal any statistically significant differences between specific pairs of educational groups.

**Table 1:** Baseline characteristics of participants with or without PSU (*N* = 732).

Variables	Total (*N* = 732)	PSU (*N* = 358)	Non-PSU (*N* = 374)	Statistics	*P*-value
Age (years)	22.98 ± 4.68	22.59 ± 4.59	23.36 ± 4.73	6.029	0.014^*^
Gender					
Male	348 (47.5%)	188 (52.5%)	160 (42.8%)	6.948	0.008^**^
Female	384 (52.5%)	170 (47.5%)	214 (57.2%)		
Educational attainment					
Undergraduate	529 (72.3%)	276 (77.1%)	253 (67.6%)	8.248	0.016^*^
Postgraduate	141 (19.3%)	58 (16.2%)	83 (22.2%)		
Doctoral student	62 (8.5%)	24 (6.7%)	38 (10.2%)		
Study major					
Liberal arts and economics	609 (83.2%)	298 (83.2%)	311 (83.2%)	0.001	0.975
Science and medicine	123 (16.8%)	60 (16.8%)	63 (16.8%)		
Living arrangement					
Alone	644 (88.0%)	317 (88.5%)	327 (87.4%)	0.215	0.643
Joint rent	88 (12.0%)	41 (11.5%)	47 (12.6%)		
Daily smartphone use					
< 2 h	125 (17.1%)	47 (13.1%)	78 (20.9%)	15.98	< 0.001^***^
2–4 h	497 (67.9%)	241 (67.3%)	256 (68.4%)		
≥5 h	110 (15.0%)	70 (19.6%)	40 (10.7%)		
Duration of study aboard					
< 1 years	242 (33.1%)	118 (33.0%)	124 (33.2%)	0.351	0.839
1–3 years	345 (47.1%)	166 (46.4%)	179 (47.9%)		
>3 years	145 (19.8%)	74 (20.7%)	71 (19.0%)		
Marital status					
Unmarried	688 (94.0%)	336 (93.9%)	352 (94.1%)	0.022	0.881
Married	44 (6.0%)	22 (6.1%)	22 (5.9%)		
JLPT level					
N2	571 (78.0%)	279 (77.9%)	292 (78.1%)	0.002	0.963
N1	161 (22.0%)	79 (22.1%)	82 (21.9%)		
Nationality^a^					
East Asia	505 (69.0%)	256 (71.5%)	249 (66.6%)	3.683	0.159
Southeastern Asia	183 (25.0%)	86 (24.0%)	97 (25.9%)		
Others	44 (6.0%)	16 (4.5%)	28 (7.5%)		
Having relative in Japan					
No	637 (87.0%)	306 (85.5%)	331 (88.5%)	1.485	0.223
Yes	95 (13.0%)	52 (14.5%)	43 (11.5%)		
Living expense sources					
Scholarship	51 (7.0%)	25 (7.0%)	26 (7.0%)	0.413	0.938
Family	594 (81.1%)	288 (80.4%)	306 (81.8%)		
Loans/personals saving	65 (8.9%)	33 (9.2%)	32 (8.6%)		
Others	22 (3.0%)	12 (3.4%)	10 (2.7%)		

Continuous variables are presented as mean ± standard deviation, and categorical variables as n (%). Between-group differences were examined using independent-samples t-tests for continuous variables and Pearson's chi-square tests for categorical variables.

^a^East Asia included China, Mongolia, and South Korea; Southeastern Asia included Vietnam, Thailand, Myanmar, and Sri Lanka.

JLPT, Japanese language proficiency test; PSU, problematic smartphone use.

^*^P < 0.05, ^**^P < 0.01, ^***^P < 0.001.

### 3.2 Pearson correlation analysis

Table 2 presents the means (SD) and Pearson correlations among the study variables. The results revealed that acculturative stress demonstrated a significant positive correlation with expressive suppression (*r* = 0.467, *P* < 0.01), social anxiety (*r* = 0.386, *P* < 0.01), and PSU (*r* = 0.544, *P* < 0.01), while exhibiting a significant negative correlation with cognitive reappraisal (*r* = −0.360, *P* < 0.01). Furthermore, expressive suppression was positively associated with social anxiety (*r* = 0.327, *P* < 0.01) and PSU (*r* = 0.405, *P* < 0.01). In contrast, cognitive reappraisal was negatively related to expressive suppression (*r* = −0.215, *P* < 0.01), social anxiety (*r* = −0.396, *P* < 0.01), and PSU (*r* = −0.573, *P* < 0.01). Furthermore, there was a significant positive relationship between social anxiety and PSU (*r* = 0.656, *P* < 0.01). These bivariate correlations are provided for preliminary descriptive purposes to provide an overview of the initial associations among variables before proceeding to the more rigorous SEM analysis.

**Table 2:** Descriptive statistics and correlations of study variables (*N* = 732).

Variables	Mean	SD	1.	2.	3.	4.
1. Acculturative stress	91.81	28.85	1.000	-	-	-
2. Cognitive reappraisal	17.89	6.06	−0.360^**^	1.000	-	-
3. Expressive suppression	18.44	5.08	0.467^**^	−0.215^**^	1.000	-
4. Social anxiety	44.69	15.27	0.386^**^	−0.396^**^	0.327^**^	1.000
5. PSU	33.10	11.43	0.544^**^	−0.573^**^	0.405^**^	0.656^**^

PSU, problematic smartphone use.

^**^P < 0.01.

### 3.3 Structural model and mediation analysis

The CFA supported the adequacy of the measurement model. All standardized factor loadings were statistically significant and exceeded 0.50, ranging from 0.692 to 0.846. CR values ranged from 0.721 to 0.951, and AVE values ranged from 0.514 to 0.662. All HTMT ratios were below 0.85, supporting adequate discriminant validity among the constructs. Detailed reliability, convergent validity, and discriminant validity results are presented in Tables 3, 4.

**Table 3:** Construct reliability and validity of the structured model.

Construct	Subscale (no. of items)	Items	Factor loading	Cronbach's alpha	CR	AVE	χ^2^/df	GFI	IFI	TLI	CFI	RMSEA	SRMR
SAS-SV	— (10)	SA1	0.807	0.951	0.951	0.662	1.668	0.984	0.996	0.995	0.996	0.030	0.013
		SA2	0.801										
		SA3	0.817										
		SA4	0.817										
		SA5	0.802										
		SA6	0.784										
		SA7	0.813										
		SA8	0.824										
		SA9	0.824										
		SA10	0.846										
ASSIS	PD (8)	PD1	0.715	0.900	0.900	0.531	1.130	0.954	0.996	0.995	0.996	0.013	0.017
		PD2	0.733										
		PD3	0.719										
		PD4	0.72										
		PD5	0.761										
		PD6	0.717										
		PD7	0.716										
		PD8	0.743										
	HM (4)	HM1	0.739	0.832	0.832	0.553							
		HM2	0.740										
		HM3	0.748										
		HM4	0.747										
	PH (5)	PH1	0.737	0.850	0.850	0.531							
		PH2	0.721										
		PH3	0.705										
		PH4	0.736										
		PH5	0.743										
	FE (4)	FE1	0.728	0.809	0.810	0.515							
		FE2	0.709										
		FE3	0.700										
		FE4	0.734										
	SC (3)	SC1	0.736	0.784	0.784	0.548							
		SC2	0.742										
		SC3	0.742										
	GU (2)	GU1	0.748	0.721	0.721	0.564							
		GU2	0.755										
	NC (10)	NC1	0.724	0.913	0.913	0.514							
		NC2	0.723										
		NC3	0.706										
		NC4	0.717										
		NC5	0.692										
		NC6	0.731										
		NC7	0.718										
		NC8	0.724										
		NC9	0.710										
		NC10	0.721										
ERQ	ERQC (6)	CR1	0.752	0.864	0.864	0.514	1.107	0.995	0.999	0.999	0.999	0.012	0.013
		CR2	0.695										
		CR3	0.730										
		CR4	0.693										
		CR5	0.716										
		CR6	0.715										
	ERQE (4)	ES1	0.756	0.846	0.846	0.578	0.872	0.999	1.000	1.001	1.000	< 0.001	0.007
		ES2	0.746										
		ES3	0.779										
		ES4	0.760										
LSAS	SAX (11)	SAX1	0.736	0.927	0.927	0.535	1.151	0.969	0.997	0.996	0.997	0.014	0.017
		SAX2	0.731										
		SAX3	0.735										
		SAX4	0.734										
		SAX5	0.728										
		SAX6	0.741										
		SAX7	0.720										
		SAX8	0.730										
		SAX9	0.746										
		SAX10	0.739										
		SAX11	0.706										
	SAB (13)	SAB1	0.744	0.937	0.937	0.533							
		SAB2	0.741										
		SAB3	0.726										
		SAB4	0.741										
		SAB5	0.714										
		SAB6	0.743										
		SAB7	0.721										
		SAB8	0.730										
		SAB9	0.704										
		SAB10	0.735										
		SAB11	0.736										
		SAB12	0.724										
		SAB13	0.730										

SAS-SV, smartphone addiction scale—short version; ASSIS, acculturative stress scale for international students; PD, perceived discrimination; HM, homesickness; PH, perceived hate/rejection; FE, fear; SC, stress due to culture shock/change; GU, guilt; NC, nonspecific concerns; ERQ, emotion regulation questionnaire; ERQC, cognitive reappraisal; ERQE, expressive suppression; LSAS, Liebowitz social anxiety scale; SAX, social anxiety—fear; SAB, social anxiety—avoidance behavior; CR, composite reliability; AVE, average variance extracted; χ^2^/df, chi-square/degrees of freedom ratio; GFI, goodness of fit index; IFI, incremental fit index; TLI, Tucker-Lewis index; CFI, comparative fit index; RMSEA, root mean square error of approximation; SRMR, standardized root mean square residual.

**Table 4:** Discriminant validity (HTMT ratio).

Construct	ERQC	ERQE	LSAS	ASSIS
ERQC				
ERQE	0.251			
LSAS	0.435	0.362		
ASSIS	0.394	0.512	0.401	
SAS-SV	0.632	0.451	0.686	0.566

Values represent the Heterotrait-Monotrait (HTMT) ratio of correlations.

SAS-SV, smartphone addiction scale—short version; ASSIS, acculturative stress scale for international students; ERQC, cognitive reappraisal; ERQE, expressive suppression; LSAS, Liebowitz social anxiety scale.

After adjusting for control variables including gender, age, educational attainment, and daily smartphone use, the structural model yielded an excellent fit to the data: χ^2^/df = 1.975, RMSEA = 0.037, SRMR = 0.051, GFI = 0.986, NFI = 0.963, CFI = 0.981, and TLI = 0.971.

**Figure 3:**
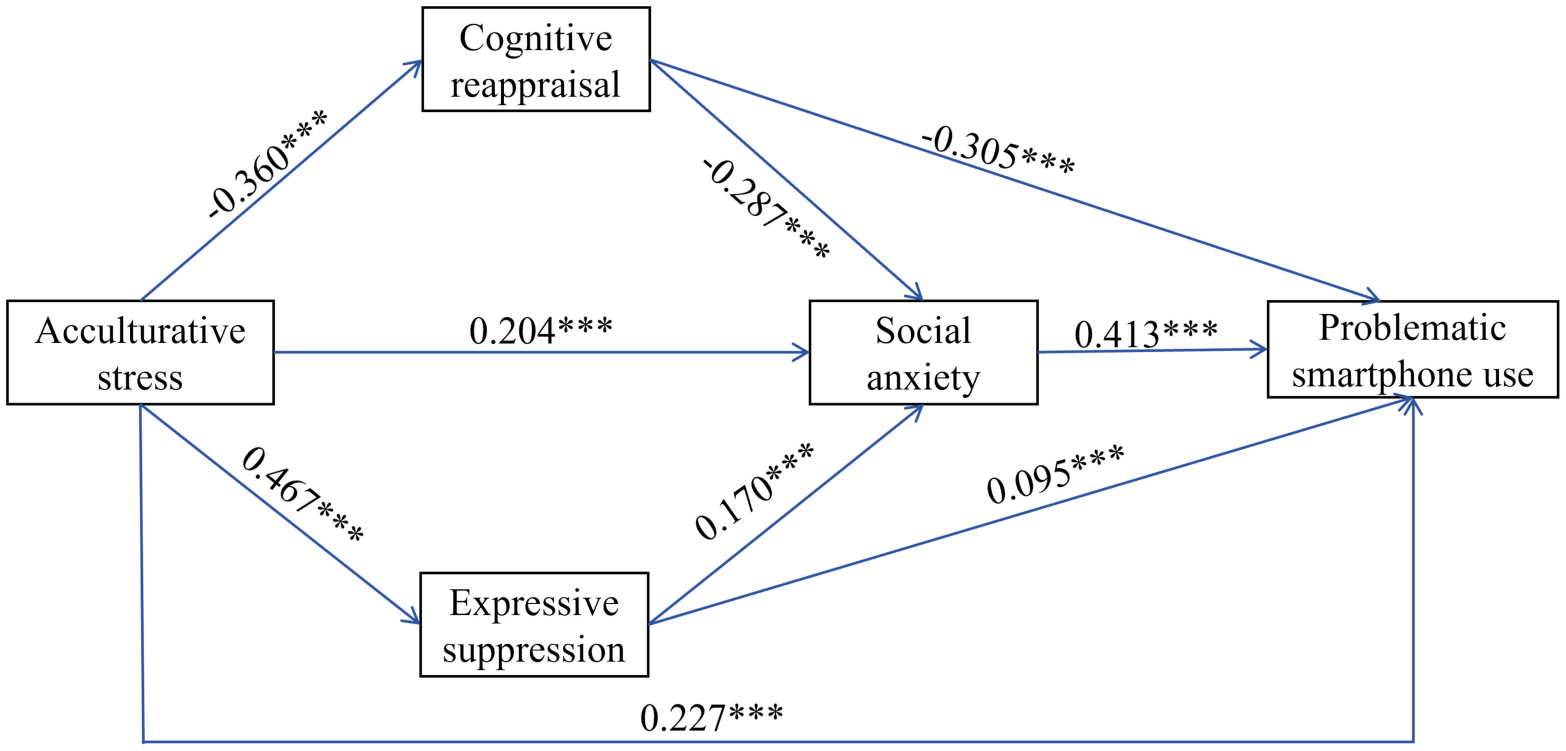
Chain mediation model of the association between acculturative stress and problematic smartphone use through emotion regulation and social anxiety. The values presented on the paths represent standardized path coefficients (β). The significance of direct pathways was evaluated based on standardized estimates. ***P < 0.001.

Mediation hypotheses were tested using structural equation modeling in AMOS 26.0. As illustrated in Figure 3 and Table 5, all structural paths were statistically significant. The coefficients reported in Figure 3 and Table 5 are standardized path coefficients (β). Aacculturative stress exhibited a significant positive effect on expressive suppression (β = 0.467, *P* < 0.001), social anxiety (β = 0.204, *P* < 0.001), and PSU (β = 0.227, *P* < 0.001). Conversely, acculturative stress demonstrated a significant negative effect on cognitive reappraisal (β = −0.360, *P* < 0.001). Expressive suppression was positively related to social anxiety (β = 0.170, *p* < 0.001) and PSU (β = 0.095, *P* < 0.001). Social anxiety further showed a strong positive relationship with PSU (β = 0.413, *P* < 0.001). In contrast, cognitive reappraisal was inversely associated with social anxiety (β = −0.287, *P* < 0.001) and PSU (β = −0.305, *P* < 0.001).

**Table 5:** Structural model results: standardized path coefficients and statistical estimates.

Path	β	SE	*P*-value	95% CI
Acculturative stress → cognitive reappraisal	−0.360	0.035	< 0.001	(−0.426, −0.289)
Acculturative stress → expressive suppression	0.467	0.023	< 0.001	(0.419, 0.511)
Acculturative stress → social anxiety	0.204	0.038	< 0.001	(0.129, 0.278)
Acculturative stress → PSU	0.227	0.030	< 0.001	(0.167, 0.288)
Cognitive reappraisal → social anxiety	−0.287	0.034	< 0.001	(−0.353, −0.220)
Cognitive reappraisal → PSU	−0.305	0.030	< 0.001	(−0.364, −0.247)
Expressive suppression → social anxiety	0.170	0.038	< 0.001	(0.097, 0.243)
Expressive suppression → PSU	0.095	0.026	< 0.001	(0.045, 0.147)
Social anxiety → PSU	0.413	0.031	< 0.001	(0.350, 0.473)

β represents the standardized path coefficient. SE, P values, and 95% CIs correspond to the standardized coefficients.

CI, confidence interval; SE, standard error; PSU, problematic smartphone use.

The mediating roles of emotion regulation strategies and social anxiety in the association between acculturative stress and PSU were tested using a bootstrapping procedure. The total, direct, and indirect effects reported in Table 6 are unstandardized estimates. The total effect of acculturative stress on PSU was significant (*B* = 0.211, *P* = 0.001). After accounting for the mediators, the direct effect remained significant (*B* = 0.089, *P* < 0.001), accounting for 42.2% of the total effect. The total indirect effect was also significant (*B* = 0.123, *P* < 0.001), accounting for 58.3% of the total effect and indicating partial mediation. Five specific indirect pathways were statistically significant. These included three simple mediation pathways through cognitive reappraisal (*B* = 0.043, *P* < 0.001), expressive suppression (*B* = 0.017, *P* < 0.001), and social anxiety (*B* = 0.033, *P* < 0.001), as well as two serial mediation pathways through cognitive reappraisal and social anxiety (*B* = 0.017, *P* < 0.001) and through expressive suppression and social anxiety (*B* = 0.013, *P* < 0.001). Because none of the bootstrap 95% confidence intervals included zero, all five indirect effects were statistically significant. These findings indicate that acculturative stress was associated with PSU both directly and indirectly through emotion regulation strategies and social anxiety.

**Table 6:** Unstandardized direct and indirect effects of the mediation model.

Paths	Estimate	SE	*P*-value	Bootstrap 95% CI		Proportion
	(B)			LLCI	ULCI	
Total effect	0.211	0.015	0.001	0.180	0.239	
Direct effect	0.089	0.012	< 0.001	0.064	0.113	42.2%
Total indirect effect	0.123	0.010	< 0.001	0.104	0.142	58.3%
Acculturative stress → cognitive reappraisal → PSU	0.043	0.006	< 0.001	0.032	0.055	20.4%
Acculturative stress → expressive suppression → PSU	0.017	0.005	< 0.001	0.008	0.028	8.1%
Acculturative stress → social anxiety → PSU	0.033	0.007	< 0.001	0.020	0.047	15.6%
Acculturative stress → cognitive reappraisal → social anxiety → PSU	0.017	0.003	< 0.001	0.012	0.023	8.1%
Acculturative stress → expressive suppression → social anxiety → PSU	0.013	0.003	< 0.001	0.007	0.020	6.2%

All direct, indirect, and total effects are reported as unstandardized coefficients.

B, unstandardized effect estimate; CI, confidence interval; LLCI, lower limit confidence interval; ULCI, upper limit confidence interval; SE, standard error; PSU, problematic smartphone use.

## 4 Discussion

### 4.1 Main findings

This study examined the associations among acculturative stress, cognitive reappraisal, expressive suppression, social anxiety, and PSU among international students in Japan. Acculturative stress was associated with higher PSU both directly and indirectly through the three proposed mediators. The results also supported two sequential pathways in which cognitive reappraisal or expressive suppression was associated with social anxiety, which was subsequently related to PSU. Overall, all six hypotheses were supported. These findings suggest that PSU among international students should be considered within the broader context of cross-cultural adaptation, emotional coping, and interpersonal adjustment rather than as an isolated pattern of excessive technology use.

### 4.2 Associations between acculturative stress and PSU

The 48.9% prevalence of PSU in our sample aligns with findings among international students in China (41) but is higher than rates reported in South Korea (42), likely reflecting measurement and contextual variations. Supporting H1, we confirmed a significant direct relationship between acculturative stress and PSU, consistent with prior literature (7, 8). International students frequently experience language barriers, homesickness, discrimination, academic demands, and difficulties establishing supportive relationships in the host country (43). Smartphones may provide immediate access to familiar social networks, practical information, distraction, and emotional relief (44). Although these functions can be beneficial, frequent reliance on smartphones as a primary coping strategy may be associated with impaired control and interference with academic or social functioning (45). This interpretation is consistent with Compensatory Internet Use Theory, which proposes that excessive digital engagement may occur in response to psychosocial distress or unmet offline needs (6, 16).

### 4.3 Mediating and sequential psychological pathways

Supporting H2 and H3, cognitive reappraisal and expressive suppression significantly mediated the acculturative stress–PSU link. Chronic cross-cultural stress often impairs individuals' capacity to adaptively reframe challenging experiences, prompting increased reliance on smartphones for coping (46). Conversely, the increased use of expressive suppression—often employed to maintain interpersonal harmony in East Asian cultural contexts—can leave internal distress unresolved, fostering avoidance-oriented digital coping (47, 48). Notably, cognitive reappraisal exhibited a stronger mediating effect than suppression. This suggests that the adaptive reinterpretation of stressors provides a more robust buffer against behavioral addictions than simply inhibiting emotional expression, emphasizing its utility across cultural contexts (9).

Social anxiety also significantly mediated the association between acculturative stress and PSU, supporting H4. International students may experience heightened fear of negative evaluation because of unfamiliar communication norms, limited language confidence, or concern about making interpersonal mistakes (49). Smartphone-mediated communication may feel safer because it provides greater control and fewer immediate social demands (50). However, repeated reliance on digital interaction may reinforce avoidance and reduce opportunities to develop confidence in face-to-face settings (51). This interpretation is consistent with previous evidence linking social anxiety with PSU (52, 53).

The results also support the hypothesized sequential mediations (H5 and H6), demonstrating that acculturative stress influences PSU through emotion regulation and subsequent social anxiety. These pathways suggest that difficulties in emotional coping may be linked to greater interpersonal anxiety during cultural adaptation, while social anxiety may increase reliance on smartphone-mediated interaction. In the first pathway (H5), diminished cognitive reappraisal limits a student's ability to positively reframe cultural challenges, which directly intensifies social anxiety and digital avoidance (54). In the second pathway (H6), utilizing expressive suppression to align with host-culture norms limits genuine interpersonal engagement and fosters hypervigilance toward rejection (55, 56). Consequently, elevated social anxiety drives students to use smartphones as a “digital shield” against uncomfortable face-to-face interactions (57).

### 4.4 Research significance and global public health implications

This study provides important implications for understanding and addressing PSU among international students. Rather than considering PSU solely as excessive technology use, the findings suggest that it should be examined within the broader context of acculturative stress, emotion regulation, and social anxiety. The identified mediating pathways highlight potential targets for intervention beyond simple screen-time reduction.

From a public health perspective, university support services may benefit from integrating digital well-being promotion with existing psychological and academic support programs. Interventions that enhance adaptive emotion regulation, reduce social anxiety, and facilitate offline social connection may help international students develop healthier coping strategies during cultural adaptation.

Although this study was conducted among international students in Japan, the proposed psychosocial framework may provide a useful reference for other higher education settings with culturally diverse student populations. Future longitudinal and intervention studies are needed to further evaluate the effectiveness of culturally sensitive approaches for promoting mental well-being and healthy digital behaviors.

### 4.5 Limitations and future prospects

Several limitations should be considered. First, the cross-sectional design does not establish temporal order or causality; longitudinal and prospective studies are needed to examine whether the proposed pathways unfold over time. Second, all variables were measured by self-report, which may introduce recall, social desirability, and common method bias. Future studies could incorporate objective smartphone use records, clinical assessments, or reports from multiple sources. Third, convenience sampling and the predominance of participants from Asian backgrounds may limit generalizability to other international student populations. Fourth, potentially relevant factors, including resilience, academic stress, social support, and pre-existing mental health conditions, were not included in the model. Finally, although validated Japanese measures were used where available and the remaining scales underwent forward–backward translation and pilot testing, formal cross-language measurement invariance was not examined. Future research should evaluate the model in more culturally diverse samples and test the equivalence of translated measures across language groups.

## 5 Conclusion

This study empirically tested and supported a six-hypothesis conceptual model linking acculturative stress with PSU among international students in Japan. Cognitive reappraisal, expressive suppression, and social anxiety statistically accounted for significant portions of this association, both independently and sequentially. By contextualizing this phenomenon within a non-Western host country, this research expands the current epidemiological understanding of acculturation and behavioral addictions. From a public health and institutional policy perspective, these results highlight the critical need for targeted, university-led interventions. Campus health initiatives should prioritize cultivating adaptive emotion regulation skills, mitigating social anxiety, and facilitating meaningful offline social integration to prevent PSU and protect the broader mental well-being of vulnerable international student populations.

## Data Availability

The raw data supporting the conclusions of this article will be made available by the authors, without undue reservation.
